# rTMS combined with median nerve magnetic stimulation for prolonged disorders of consciousness following intracerebral hemorrhage: a randomized controlled trial protocol

**DOI:** 10.3389/fneur.2025.1671226

**Published:** 2025-10-24

**Authors:** Hanbo Chen, Si Chen, Weifeng Wen, Yongliang Guo, Yong Luo, Junfu Li, Shujuan Huang, Xiao Lv

**Affiliations:** ^1^Department of Rehabilitation Therapy, Guangdong Sanjiu Brain Hospital, Guangzhou, China; ^2^Department of Rehabilitation Medicine, Guangdong Sanjiu Brain Hospital, Guangzhou, China

**Keywords:** rTMS, median nerve magnetic stimulation, intracerebral hemorrhage, prolonged disorders of consciousness, protocol

## Abstract

**Background:**

Prolonged disorders of consciousness (pDoC) following intracerebral hemorrhage significantly impact patient quality of life, with limited effective standardized treatments available. While repetitive transcranial magnetic stimulation (rTMS) and median nerve stimulation show individual therapeutic potential, high-quality clinical evidence for combined treatment protocols remains lacking.

**Methods:**

This randomized controlled trial will enroll 129 patients with pDoC following intracerebral hemorrhage, randomly allocated to three groups: combined group (median nerve magnetic stimulation (MNMS) followed by rTMS treatment), rTMS group (sham MNMS followed by rTMS treatment), MNMS group (MNMS followed by sham rTMS treatment). The primary outcome is the Coma Recovery Scale-Revised (CRS-R) score at 3 weeks post-treatment. Secondary outcomes include Glasgow Coma Scale scores, brainstem auditory evoked potentials, somatosensory evoked potentials, and safety assessments. Statistical analysis will employ repeated measures ANOVA and appropriate post-hoc tests.

**Discussion:**

The combined treatment mechanism is based on multilevel consciousness network modulation theory, integrating “top-down” cortical regulation through rTMS and “bottom-up” sensory pathway activation through median nerve magnetic stimulation. This bidirectional approach may achieve more comprehensive consciousness network repair compared to single modalities. The study’s rigorous three-group design and comprehensive assessment combining clinical scales with electrophysiological indicators will provide robust evidence for the clinical application of neuromodulation techniques in consciousness disorders.

**Clinical trial registration:**

https://www.chictr.org.cn/showproj.html?proj=256274, identifier ChiCTR2500106064.

## Introduction

Prolonged disorders of consciousness (pDoC) following intracerebral hemorrhage (ICH) refer to abnormal levels of consciousness and arousal that persist beyond 28 days post-injury, primarily encompassing vegetative state/unresponsive wakefulness syndrome (VS/UWS) and minimally conscious state (MCS) (1). This condition results in consciousness impairment through disruption of critical neural networks, including the brainstem reticular activating system and thalamo-cortical circuits (2, 3).

The global annual incidence of ICH is approximately 24.6 per 100,000 person-years, with approximately 15–20% of ICH survivors developing pDoC (4). Among these patients, 40–60% remain in a state of consciousness disorder after 1 year (5), imposing substantial burdens on families and society.

Current therapeutic approaches primarily include pharmacological treatments, physical therapy, and neuromodulation techniques. However, existing treatment modalities exhibit significant limitations: pharmacological interventions demonstrate low efficacy rates and are associated with adverse effects (6, 7); physical rehabilitation lacks standardized protocols (1, 8); and invasive neuromodulation techniques carry high surgical risks and substantial costs (9, 10).

Non-invasive neuromodulation techniques offer advantages of safety, non-invasiveness, and cost-effectiveness. Recent systematic reviews and meta-analyses have provided increasingly robust evidence for rTMS efficacy in consciousness disorders. A recent meta-analysis analyzed 17 randomized controlled trials encompassing 377 patients and demonstrated moderate-quality evidence supporting rTMS for improving consciousness levels (11). However, substantial heterogeneity existed in treatment protocols, with stimulation frequencies ranging from 5 to 20 Hz, intensities from 80 to 120% resting motor threshold (RMT), and treatment durations from 2 to 8 weeks, making clinical standardization challenging. Network meta-analysis that while various neuromodulation techniques showed efficacy, most trials examined single-modality interventions, with limited high-quality evidence for synergistic effects of combined central-peripheral stimulation approaches (12). The synergistic combination of central nervous system neuromodulation with peripheral nerve stimulation can enhance brain plasticity, effectively promote brain functional recovery, and improve consciousness levels, demonstrating theoretical superiority over single-modality stimulation (13). Studies have demonstrated that the synergistic modulation combining transcranial magnetic stimulation (TMS) with median nerve stimulation (MNS) could better enhance consciousness levels (14). However, MNS presents several limitations, including prolonged treatment duration (requiring continuous stimulation for 2–8 h daily), interference with nursing care, skin allergies, electrode displacement during perspiration, tissue damage and pain caused by high-density surface currents (15, 16). Median nerve magnetic stimulation (MNMS) is a novel stimulation modality. Beyond operational convenience, magnetic stimulation can induce a more uniform induced current at a greater depth without increasing skin current density, enhancing recruitment of the median nerve trunk and proximal afferent bundles, and eliciting stronger activation in S1 and the thalamic nonspecific nuclei (17). Previous rPMS/MNMS studies in healthy subjects and patients with chronic pain or motor disorders have demonstrated facilitation of cortical excitability and better tolerability (18, 19). For DoC populations requiring high-frequency/high-density therapy, MNMS shortens per-session duration, reduces nursing interference, and lowers the risk of skin-related adverse events (15, 16). Despite the theoretical advantages of combined neuromodulation, systematic reviews have identified critical evidence gaps. The study noted that among 28 included studies, only three examined combined central-peripheral approaches, with significant methodological limitations including absence of sham controls and inadequate sample sizes. Furthermore, most studies used prolonged electrical stimulation protocols (4–8 h daily), which present practical implementation challenges in clinical settings (12). Our study addresses these gaps by employing magnetic rather than electrical peripheral stimulation, implementing a rigorous three-group sham-controlled design, and using clinically feasible treatment durations. Preliminary case observations revealed that TMS combined with MNMS could significantly enhance brain functional network connectivity in patients with pDoC following ICH. However, pDoC has diverse causes with varying damage patterns. ICH, 10–15% of strokes, directly injures subcortical structures (basal ganglia, thalamus) and white-matter tracts while sparing most cortex, unlike diffuse axonal trauma or laminar cortical necrosis after hypoxia. Because ICH mainly disrupts thalamo-reticular arousal systems, these patients may respond well to combined “top-down” cortical plus “bottom-up” peripheral stimulation. Therefore, we will test rTMS plus MNMS versus either alone in ICH-related pDoC for clinical and neurophysiological efficacy.

### Study design

**Figure 1:**
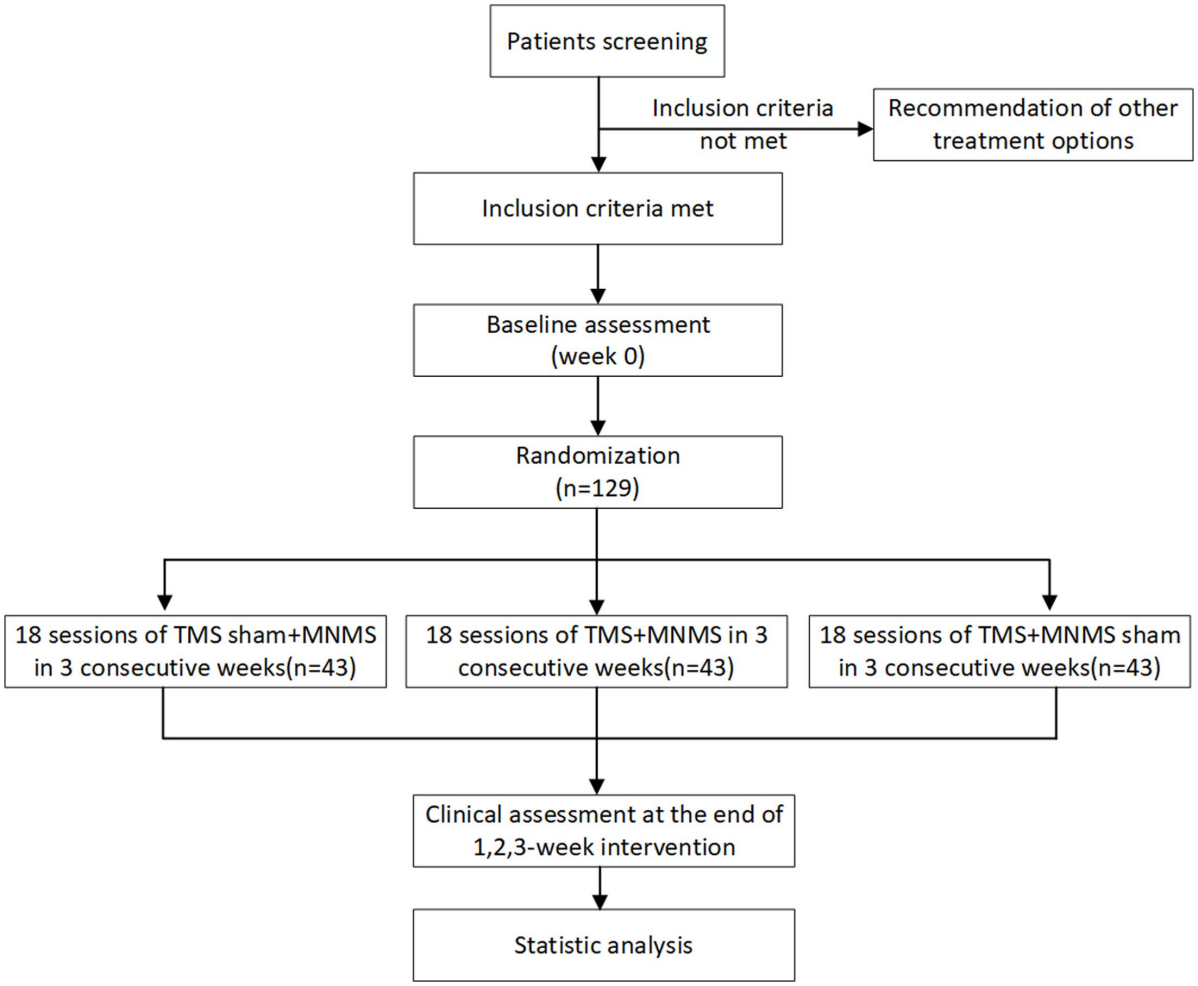
Study design (randomized controlled trials) and assessment time points (consort chart).

This study employs a single-center, parallel-controlled, randomized controlled trial design with a study period of 24 months, conducted at the Rehabilitation Medicine Center of Guangdong Sanjiu Brain Hospital. The study is expected to recruit 129 patients with pDoC following ICH. This research has been approved by the Ethics Committee of Guangdong Sanjiu Brain Hospital. The flow diagram of study is shown in Figure 1.

### Inclusion criteria

Patients with pDoC caused by ICH, with diagnosis conforming to the Practice Guidelines for Disorders of Consciousness (1); diagnosed as VS or MCS according to the Coma Recovery Scale-Revised (CRS-R), with CRS-R scores of 4–15 points (20, 21) and Glasgow Coma Scale (GCS) scores of 4–12 points (1); disease duration of 3–6 months, first episode of ICH with stable clinical symptoms, without mechanical ventilation support; absence of significant cerebral edema and severe cerebral atrophy; age 18–70 years; stimulation sites without skull defects, infection, bleeding points, or damage; legal guardian consent and signed informed consent form.

### Exclusion criteria

Presence of treatment contraindications such as pacemakers, history of cranioplasty, metallic brain implants, or neurostimulators; patients currently taking sedative medications and Na+ or Ca2+ channel blockers or NMDA receptor antagonists; patients currently undergoing neuromodulation treatments such as TMS or vagus nerve stimulation; previous history of epilepsy, psychiatric disorders, neurodegenerative diseases, or history of substance abuse and alcohol abuse (defined as documented substance use disorder diagnosis or addiction treatment within 2 years prior to ICH onset; patients with alcohol as an indirect ICH risk factor such as alcohol-induced hypertension who do not meet abuse/dependence criteria are not excluded) (22–24); presence of other diseases causing consciousness disorders, such as severe acute infections, endocrine and metabolic disorders, space-occupying brain lesions, or water-electrolyte imbalances; patients with tendency for recurrent ICH; presence of factors affecting consciousness alteration, such as hyperglycemia or hypoglycemia, electrolyte disturbances, or infections; concurrent participation in other interventional clinical trials, as well as patients deemed unsuitable for participation in this study by the investigators.

### Sample size calculation

This study employed a one-way analysis of variance (ANOVA) *F*-test power analysis to determine the sample size for comparing continuous outcomes across three independent groups. We began with a recent meta-analysis of rTMS in pDoC reporting a weighted mean difference (WMD) of 1.89 CRS-R points between intervention and control groups (25). To ensure our trial is powered to detect clinically meaningful improvements, we first defined the minimal clinically important difference (MCID) for CRS-R. Based on the probabilistic analysis by Monti et al. (26), which employed Bayesian modeling and expert consensus specifically for DoC populations, a 2-point CRS-R change represents the MCID (26). Drawing on pooled standard deviations of approximately 4.5 points from prior clinical trials in pDoC, we calculated the target effect size as Cohen’s *d* = 2.0/4.5 ≈ 0.44. Under standard assumptions for three-group comparisons, this corresponds to an ANOVA effect size of approximately *f* ≈ 0.22. Considering the reported heterogeneity of effects in pDoC neuromodulation (commonly spanning *f* = 0.20–0.40 (9, 14, 27)) and the potential for enhanced efficacy with the combined intervention relative to single-modality treatment, we conservatively prespecified a moderate effect size of *f* = 0.30 for sample size planning, which provides adequate power to detect group differences at or exceeding the established 2-point MCID threshold.

Statistical parameters were set as follows: *a* = 0.05 (two-sided), power 1−*β* = 0.80, and equal allocation across three groups (1:1:1). Using G*Power 3.1.9.7 (University of Düsseldorf, Germany), the required sample size was 36 participants per group (108 total) to detect the prespecified effect size. To address anticipated attrition in pDoC interventional trials, we incorporated a 15% dropout adjustment (supported by recent trials and reviews) (28, 29)., yielding a target of 43 participants per group and a total planned enrollment of 129 participants. This design maintains adequate statistical power to detect clinically meaningful differences in CRS-R across treatment groups while preserving study feasibility.

### Randomization and grouping

An adaptive minimization randomization strategy combined with block randomization was employed, with algorithms constructed using R software by an independent biostatistics center. Balancing factors included age, hemorrhage location, disease duration, and baseline CRS-R scores. Researchers input patient information through a secure network platform, with the system providing real-time group allocation results, utilizing encrypted transmission to ensure allocation sequence confidentiality. Allocation concealment was achieved through a central randomization system, with the assessment team receiving only patient identification numbers and evaluation schedules. This study employed a double-blind design for assessors and patients. To control operational bias, standard operating procedures were established, treatment personnel received unified training, and a quality monitoring system was implemented.

Patients were randomly allocated to three groups: on the basis of conventional rehabilitation therapy, the combined group received rTMS combined with MNMS; the rTMS group received rTMS plus sham MNMS; the MNMS group received sham rTMS plus MNMS. This active-controlled design enables evaluation of the synergistic effects of combined treatment and the independent effects of single treatment modalities.

### Intervention measures (rTMS combined with MNMS treatment)

Patients will receive three treatment protocols in addition to conventional rehabilitation therapy: combined group (MNMS followed by rTMS treatment), rTMS group (sham MNMS followed by rTMS treatment), and MNMS group (MNMS followed by sham rTMS treatment):

1) Conventional treatment: Including conventional pharmacological treatment, limb motor therapy, standing training, low-frequency electrical stimulation therapy, swallowing function therapy, acupuncture therapy, etc., administered once daily for approximately 3 h total, with treatment provided 5 days per week.
2) MNMS treatment: The MNMS stimulation device utilized was YDR CCY-I (Yiruide, Wuhan, China) with a circular coil. During treatment, the coil was positioned parallel (handle perpendicular to forearm) at the median nerve point, 2 cm above the palmar wrist crease on the right wrist joint. Right-sided stimulation was selected based on: (1) right-hand dominance in >90% of the population, ensuring consistent sensory-motor cortical representation; (2) evidence that unilateral median nerve stimulation activates bilateral thalamic non-specific nuclei and brainstem reticular formation through crossed and uncrossed ascending pathways, providing non-lateralized arousal enhancement (17). Patients were positioned supine with the right forearm placed in pronation. Stimulation frequency was 20 Hz, with stimulation intensity set to the minimum intensity capable of inducing obvious contraction of wrist-hand muscles, delivering a total of 4,000 pulses over a total stimulation duration of 10 min. For sham MNMS treatment, the handle direction remained unchanged while the coil was rotated 90° and positioned over the right median nerve, with all other parameters identical to active stimulation.
3) rTMS treatment: The rTMS stimulation device utilized was YDR CCY-I (Yiruide, Wuhan, China) with a figure-8 coil. During treatment, the coil was placed tangentially over the scalp above the left dorsolateral prefrontal cortex (localized as F3 according to the 10/20 international EEG system), with the coil plane parallel to the scalp and the handle pointing posterolaterally at approximately 45° to the midline to induce a posterior–anterior current. The selection of left DLPFC was based on evidence from prior randomized controlled trials in pDoC populations, demonstrating that 10 Hz stimulation of this region enhances bilateral frontoparietal network connectivity, activates language-related cortices, and facilitates consciousness recovery regardless of lesion laterality (30). Patients were positioned supine. The RMT for the right first dorsal interosseous muscle was determined at the “hotspot” of the left primary motor cortex (M1) using the 5/10 method (motor-evoked potentials ≥ 50 μV in at least 5 out of 10 trials). Stimulation intensity was set at 100% RMT. The stimulation frequency was 10 Hz, delivering a total of 1,500 pulses over a total duration of 20 min. For sham rTMS treatment, the handle direction remained unchanged while the coil was rotated 90° and positioned at the treatment site (F3), with all other parameters identical to active stimulation.
4) Treatment course: All patients received the above-mentioned sequential treatment protocol of MNMS treatment (or sham MNMS) followed by rTMS treatment (or sham rTMS) on the basis of conventional treatment, with a treatment duration of 30 min (MNMS treatment for 10 min followed by rTMS treatment for 20 min), administered once daily, 6 days per week, for 3 consecutive weeks.

### Behavioral scale assessment

1) Coma Recovery Scale-Revised (CRS-R): The CRS-R primarily evaluates six domains in patients with disorders of consciousness, including auditory function, visual function, motor function, oromotor/verbal function, communication, and arousal. The maximum score is 23 points and the minimum score is 0 points, with higher scores indicating better levels of consciousness state. Consistent with the MCID used for sample size planning, a CRS-R improvement ≥2 points will be considered clinically meaningful based on the probabilistic analysis by Monti et al. (26), and responder rates (percentage of patients achieving ≥2-point improvement) will be compared across groups as a secondary outcome.
2) Glasgow Coma Scale (GCS): The GCS assesses patient consciousness levels through three aspects (eye opening response, verbal response, and motor response). The maximum achievable score is 15 points and the minimum achievable score is 3 points, with lower scores indicating more severe levels of consciousness impairment.

### Evoked potential examination

1) Brainstem Auditory Evoked Potential (BAEP): The NeuroCare detection system manufactured by Shanghai Nuocheng Electric Co., Ltd. was used for testing. Each ear was tested at least twice, with two recordings demonstrating high reproducibility and good symmetry selected as the final recorded results. The Hall evaluation criteria were used to grade the bilateral wave I–V latency and amplitude, which can be classified into grades I–IV, with lower grades indicating better BAEP status and higher grades indicating worse BAEP status. We will interpret BAEP grade reductions (≥1 grade) as electrophysiological improvement reflecting better brainstem auditory pathway integrity, which has been associated with improved arousal network function. Accordingly, we predefine electrophysiological responders as patients with ≥1-grade improvement from baseline.
2) Upper Limb Somatosensory Evoked Potential (USEP): The NeuroCare detection system manufactured by Shanghai Nuocheng Electric Co., Ltd. was used. Two results with good reproducibility and high reliability were selected as the final recorded results. The Judson evaluation criteria were used to grade the bilateral N20 latency and amplitude, classified into grades I–III, with grade I indicating good SEP function and grade III indicating poor SEP funlction. Similarly, for USEP, *a* ≥ 1-grade improvement will be considered clinically meaningful, indicating enhanced dorsal-column–medial-lemniscal conduction and somatosensory thalamo-cortical responsiveness. We will analyze BAEP/USEP grades as ordinal outcomes and examine correlations with CRS-R/GCS changes (31).

### Blinding

Participants (to the extent possible given their level of consciousness), outcome assessors, and statisticians are blinded to group allocation throughout the study. Treatment administrators cannot be blinded due to the operational requirements of delivering active versus sham stimulation but are strictly instructed not to disclose group assignments or discuss treatment procedures with assessors, participants, or family members. To maximize blinding effectiveness and minimize potential bias, several safeguards are implemented: (1) treatment administrators have no involvement whatsoever in outcome assessment; (2) treatment and assessment occur in physically separate locations; (3) family members are not permitted to be present during treatment sessions, preventing direct observation of stimulation characteristics; (4) outcome assessors have no access to treatment logs, schedules, or clinical notes that might reveal allocation; and (5) all participants receive identical numbers of treatment sessions and assessment schedules regardless of allocation.

### Predetermined visit schedule

The complete assessment schedule is presented in Table 1. Participants will be evaluated at screening (Week 2), baseline (Week 0), and during treatment (Weeks 1, 2, and 3). Demographic characteristics and medical history will be collected only at the first visit. Baseline assessments—including CRS-R, GCS, BAEP, and USEP—will be completed at Week 0, after which randomization and treatment initiation will occur. The primary outcome (CRS-R) will be assessed at every scheduled visit from baseline onward. Secondary outcomes will be measured at designated time points. All evaluations will be conducted in the rehabilitation assessment suite.

**Table 1:** Schedule of enrolment, interventions, and assessments.

Items	Study period				
	Screening	Baseline	Treatment		
Week	−2	0	1	2	3
Enrolment:					
Eligibility screen	×				
Signed informed consent	×				
Allocation		×			
Randomization		×			
Interventions:					
Combined group			×	×	×
rTMS group			×	×	×
MNMS group			×	×	×
Assessments:					
Primary outcome					
CRS-R		×	×	×	×
Secondary outcomes					
GCS		×	×	×	×
BAEP		×			×
USEP		×			×
Adverse events			×	×	×

× Represents data collection timepoint. rTMS, repetitive transcranial magnetic stimulation; MNMS, median nerve magnetic stimulation; CRS-R, Coma Recovery Scale-Revised; GCS, Glasgow Coma Scale; BAEP, Brainstem Auditory Evoked Potentials; USEP, Upper Limb Somatosensory Evoked Potential.

## Data collection and monitoring and research safety

### Data collection and quality control

This study employed the REDCap electronic data capture system (Version 12.0.15, Vanderbilt University, Nashville, TN, USA) to construct standardized electronic case report forms (eCRFs) with real-time data validation, automated logic checks, and audit trail capabilities. A double data entry verification mechanism and machine learning algorithms were implemented for data anomaly detection, with 100% source data verification conducted prior to database lock to ensure consistency between eCRFs and original medical records.

### Research safety monitoring

Based on the rTMS safety guidelines by Rossi et al. (32, 33), previous studies have shown that the main adverse events of rTMS include headache (10–15%), scalp discomfort (5–10%), and extremely rare seizures (<0.1%), while MNMS primarily causes local pain (19). A standardized adverse event monitoring system was established, including pre-treatment screening for contraindications, continuous monitoring of vital signs during procedures, clinical symptomatic treatment for adverse events, and reporting of serious adverse events to the ethics committee within 24 h.

## Statistical analysis

### Analysis principles

This study employed intention-to-treat (ITT) analysis as the primary analytical principle, including all randomized subjects. Per-protocol (PP) analysis was performed concurrently as a sensitivity analysis, including only subjects who completed ≥75% of treatment sessions and had no major protocol deviations. Statistical analysis followed the CONSORT statement, utilizing two-sided tests with a significance level of *α* = 0.05.

### Statistical analysis methods

Descriptive analysis: Continuous variables were described using mean ± standard deviation or median (interquartile range), while categorical variables were described using frequencies and percentages. Baseline characteristics were compared using one-way analysis of variance or Kruskal–Wallis test (continuous variables) and chi-square test or Fisher’s exact test (categorical variables).

Primary outcome analysis: Changes in CRS-R scores were analyzed using analysis of covariance (ANCOVA) with baseline CRS-R score, age, sex, and disease duration as covariates. Pairwise comparisons between the three groups employed Bonferroni correction. Mixed-effects models were used to analyze repeated measures data, with effect sizes calculated using Cohen’s *d*.

Secondary outcome analysis: Changes in GCS scores were analyzed using the same ANCOVA approach. Electrophysiological indicators as ordinal categorical variables were analyzed using logistic regression, with multiple comparisons corrected using the Holm-Bonferroni stepwise method.

Missing data handling: Multiple imputation was used to handle missing data, generating five imputed datasets. Sensitivity analysis employed worst-case/best-case scenario analysis to assess the impact of missing data.

Subgroup and sensitivity analysis: Predefined subgroups included baseline consciousness level, disease duration, and hemorrhage location. Sensitivity analyses included ITT vs. PP comparison, complete case analysis, and comparison of different imputation methods. Statistical analysis was performed using SAS 9.4 (SAS Institute Inc., Cary, NC, USA) and R 4.3.0 software.

### Ethics review and informed consent

This study has been reviewed and approved by the Medical Ethics Committee of Guangdong Sanjiu Brain Hospital (Ethics approval number: 2023-01-022), strictly adhering to the Declaration of Helsinki (2013 revision) and Good Clinical Practice (GCP) guidelines. The study protocol has been registered in the Chinese Clinical Trial Registry (Registration number: ChiCTR2500106064). Based on the special characteristics of patients with disorders of consciousness, informed consent forms were signed by legal representatives on behalf of the patients. The informed consent forms provided detailed descriptions of treatment protocols, possible adverse reactions, and patient rights, ensuring the voluntary nature of informed consent.

## Discussion

pDoC following ICH severely impact patient quality of life. While recent systematic evidence has established the efficacy of neuromodulation techniques for consciousness disorders, several critical gaps limit clinical translation (34). A meta-analysis by Dong et al. (11) revealed that rTMS significantly improves consciousness levels compared to standard care (SMD = 0.68), yet substantial heterogeneity in protocols (*I*^2^ = 62%) suggests that optimal parameters remain uncertain. Their subgroup analyses revealed that high-frequency stimulation (≥10 Hz) targeting the dorsolateral prefrontal cortex showed superior efficacy, supporting our choice of 10 Hz stimulation at the F3 position. However, effect sizes varied considerably across studies (range: 0.12–1.34), potentially reflecting differences in patient populations, stimulation parameters, and adjunctive therapies. It has been identified through network meta-analysis that combined approaches theoretically offer advantages, but the paucity of high-quality trials prevents definitive conclusions (12). Only 3 of 28 included studies examined combined protocols, and none employed magnetic peripheral stimulation or adequate sham controls. Furthermore, most combined protocols utilized prolonged electrical stimulation (4–8 h daily), creating practical barriers to implementation. rTMS has demonstrated efficacy in treating disorders of consciousness by modulating cortical neuronal excitability (27), while median nerve stimulation can activate the brainstem reticular formation to promote consciousness recovery (35). However, median nerve electrical stimulation has limitations including long treatment duration and discomfort. Most importantly, whether combined treatment protocols exhibit synergistic effects compared to single treatment modalities remains unsupported by high-quality clinical evidence. Based on the current research status and clinical needs described above, we designed this randomized controlled trial to systematically evaluate the efficacy and safety of rTMS combined with MNMS for treating pDoC following ICH, providing high-quality evidence-based medical evidence for the clinical application of neuromodulation techniques.

This study employs a rigorous three-group randomized controlled design that enables clear evaluation of the additive effects of combination therapy through direct comparison between the combined treatment group and two single treatment groups. Furthermore, this study integrates clinical scales (CRS-R, GCS) with electrophysiological indicators (brainstem auditory evoked potentials, somatosensory evoked potentials). This comprehensive assessment approach provides a more complete reflection of the degree of consciousness function improvement, avoiding the one-sidedness of single indicator evaluation (31).

The combined treatment mechanism of rTMS and MNMS is based on the multilevel modulation theory of consciousness networks (36). The maintenance of consciousness depends on the integrity of the brainstem-thalamus-cortical circuit (37), and ICH damages multiple nodes of this circuit, leading to impaired consciousness function (2, 3). Combined treatment may achieve more comprehensive consciousness network repair through bidirectional modulation mechanisms of “top-down” and “bottom-up” regulation (38).

rTMS primarily functions through a “top-down” cortical modulation mechanism (39). High-frequency 10 Hz stimulation of the dorsolateral prefrontal cortex enhances neuronal excitability and activates the frontoparietal attention network (40). This cortical activation is transmitted to the thalamus and brainstem reticular formation through cortico-subcortical projections, enhancing ascending arousal system function (41). Simultaneously, rTMS-induced long-term potentiation effects promote functional reorganization and synaptic plasticity of damaged neurons (42).

MNMS exerts arousal-promoting effects through “bottom-up” sensory conduction pathways (18). Stimulation signals ascend via the dorsal column-medial lemniscal pathway to the ventral posterior lateral nucleus of the thalamus, activating the somatosensory cortex (43). More importantly, these signals activate thalamic non-specific nuclei and brainstem reticular formation through collateral projections, directly enhancing ascending arousal system activity (4).

The synergistic effects of combined treatment are manifested in multilevel network integration. Cortical-level rTMS activation and subcortical MNMS activation form bidirectional modulation circuits, enhancing the functional connectivity strength of the entire consciousness network (14, 44). This multi-target, multi-pathway synchronous intervention can overcome the limitations of single treatment modalities, achieving broader neural network recruitment and functional reconstruction.

Our study design directly addresses limitations identified in recent systematic reviews while building upon established efficacy findings. Compared to trials included in previous meta-analyses (25), our protocol employs validated parameters (10 Hz, 100% RMT) while introducing three key innovations: (1) magnetic rather than electrical peripheral stimulation, reducing treatment duration from 4–8 h to 10 min while eliminating discomfort and skin complications; (2) rigorous three-group design with active controls, enabling clear differentiation of synergistic effects from individual modality contributions; and (3) specific focus on hemorrhagic stroke etiology, addressing the heterogeneity concerns raised by systematic reviews that combined multiple etiologies. Our anticipated effect size (*f* = 0.30) is conservative compared to reported findings (25), accounting for the active-controlled design and ensuring adequate power for detecting clinically meaningful differences. Furthermore, our integration of electrophysiological outcomes (BAEP, SEP) with behavioral scales responds to recent calls for multimodal assessment approaches to improve prognostic accuracy and mechanistic understanding (31).

Despite the numerous methodological advantages of this study, several inevitable limitations remain. First, our protocol employed standardized left-hemisphere rTMS and right-sided median nerve stimulation without individualization based on lesion laterality or location. While this approach enhances protocol reproducibility and facilitates clear efficacy assessment, emerging evidence suggests that lesion-specific targeting strategies may optimize treatment outcomes. For instance, contralesional rTMS (stimulating the intact hemisphere opposite to the lesion) or ipsilesional median nerve stimulation (targeting the limb corresponding to the lesion side) may enhance neural network reorganization in patients with asymmetric damage. Although our randomization strategy balanced lesion location across groups, and our planned subgroup analyses will explore whether treatment effects differ by hemorrhage location, future studies should systematically investigate individualized, lesion-adapted stimulation protocols. Additionally, bilateral stimulation approaches (e.g., bilateral median nerve stimulation or sequential bilateral rTMS) may provide more comprehensive network modulation and warrant investigation in subsequent trials.

Second, our study specifically enrolled patients with pDoC following ICH, which limits direct generalizability to other etiologies such as traumatic brain injury (TBI) or hypoxic–ischemic encephalopathy (HIE). This design choice was deliberate to ensure population homogeneity and enable clear mechanistic interpretation, but it requires careful consideration when applying our findings to mixed pDoC populations.

ICH produces a distinct neuropathological profile characterized by mechanical destruction of deep gray matter structures (thalamus, basal ganglia), disruption of subcortical–cortical white matter tracts, and inflammatory-mediated secondary injury, while often preserving cortical cytoarchitecture (44). This pattern differs fundamentally from TBI, where diffuse axonal injury causes widespread white matter shearing and multifocal cortical–subcortical damage, and from HIE, where selective neuronal vulnerability produces predominant cortical laminar necrosis, hippocampal sclerosis, and basal ganglia calcification (45).

These etiology-specific damage patterns likely influence treatment response to neuromodulation. Recent meta-analyses demonstrate heterogeneous effects across etiologies: one network meta-analysis (12) reported differential rTMS responses, with hemorrhagic stroke patients exhibiting greater CRS-R improvements than mixed populations. The predominant subcortical involvement in ICH may render these patients particularly responsive to ‘bottom-up’ peripheral stimulation (MNMS), which directly targets thalamo-cortical arousal pathways through ascending sensory projections, while preserved cortical regions remain accessible to ‘top-down’ rTMS modulation. Conversely, TBI patients with severe diffuse axonal injury may show attenuated responses to cortical stimulation due to compromised white matter connectivity between stimulation sites and deeper brain structures (46). Similarly, HIE patients with extensive cortical damage may be less responsive to DLPFC stimulation if the target region itself has undergone laminar necrosis, though preserved subcortical structures might still benefit from peripheral sensory stimulation.

Therefore, our findings should be interpreted as specifically applicable to ICH-related pDoC and require validation in TBI and HIE populations before broader clinical implementation. Clinicians considering combined rTMS+MNMS for non-ICH patients should exercise caution and monitor responses carefully.

Third, a critical limitation is that outcomes are assessed only immediately after the 3-week treatment period, with no post-treatment follow-up. This represents a significant limitation of our study design. Prior RCTs in neuromodulation for pDoC have demonstrated that treatment effects may fade without sustained intervention. Notably, tDCS-induced improvements in consciousness levels showed diminution during follow-up periods when stimulation was discontinued (47). Similarly, decay patterns in rTMS treatment effects have been observed after cessation of the intervention (48).

The theoretical mechanisms underlying our combined treatment approach—cortical excitability modulation through rTMS and ascending sensory pathway activation through MNMS—may require ongoing or periodic stimulation to maintain the induced neuroplastic changes. Without follow-up data, we cannot determine whether the improvements observed at treatment completion represent stable functional reorganization of consciousness networks or transient neurophysiological changes dependent on continued intervention. This question is particularly important for clinical translation, as the practical value of any treatment depends not only on immediate efficacy but also on the durability of benefits.

Furthermore, while BAEP and upper-limb SEP provide objective, reproducible indices of brainstem and thalamo-cortical somatosensory pathway integrity, their sensitivity to subtle cortical information processing and large-scale network reconfiguration is limited. Recent studies suggest that combining event-related potentials (e.g., MMN, P300) or resting-state EEG connectivity/complexity metrics with evoked potentials can improve the detection of subtle changes in consciousness and prognostication. In this single-center, three-arm, short-course RCT, we prioritized bedside feasibility, standardization, blinding, and data completeness; therefore, BAEP/SEP were selected as secondary outcomes at this stage. In future studies, we plan to incorporate standardized ERP paradigms and resting-state EEG connectivity/complexity measures, together with BAEP/SEP and behavioral scales, to enhance sensitivity to micro-level changes in consciousness.

## References

[1] Giacino JT Katz DI Schiff ND Whyte J Ashman EJ Ashwal S . Practice guideline update recommendations summary: disorders of consciousness: report of the guideline development, dissemination, and implementation Subcommittee of the American Academy of neurology; the American congress of rehabilitation medicine; and the National Institute on Disability, Independent Living, and Rehabilitation Research. Neurology. (2018) 91:450–60. doi: 10.1212/WNL.0000000000005926, PMID: 30089618 PMC6139814

[2] Jang SH Chang CH Jung YJ Kim JH Kwon YH. Relationship between impaired consciousness and injury of ascending reticular activating system in patients with intracerebral hemorrhage. Stroke. (2019) 50:2234–7. doi: 10.1161/STROKEAHA.118.023710, PMID: 31181997

[3] Mofakham S Fry A Adachi J Stefancin PL Duong TQ Saadon JR . Electrocorticography reveals thalamic control of cortical dynamics following traumatic brain injury. Commun Biol. (2021) 4:1210. doi: 10.1038/s42003-021-02738-2, PMID: 34675341 PMC8531397

[4] González-Pérez A Gaist D Wallander MA McFeat G García-Rodríguez LA. Mortality after hemorrhagic stroke: data from general practice (the health improvement network). Neurology. (2013) 81:559–65. doi: 10.1212/WNL.0b013e31829e6eff, PMID: 23843467

[5] Luauté J Maucort-Boulch D Tell L Quelard F Sarraf T Iwaz J . Long-term outcomes of chronic minimally conscious and vegetative states. Neurology. (2010) 75:246–52. doi: 10.1212/WNL.0b013e3181e8e8df, PMID: 20554940

[6] Giacino JT Whyte J Bagiella E Kalmar K Childs N Khademi A . Placebo-controlled trial of amantadine for severe traumatic brain injury. N Engl J Med. (2012) 366:819–26. doi: 10.1056/NEJMoa1102609, PMID: 22375973

[7] Barra ME Solt K Yu X Edlow BL. Restoring consciousness with pharmacologic therapy: mechanisms, targets, and future directions. Neurotherapeutics. (2024) 21:e00374. doi: 10.1016/j.neurot.2024.e00374, PMID: 39019729 PMC11452330

[8] Murtaugh B Morrissey A-M Fager S Knight HE Rushing J Weaver J. Music, occupational, physical, and speech therapy interventions for patients in disorders of consciousness: an umbrella review. NeuroRehabilitation. (2024) 54:109–27. doi: 10.3233/NRE-230149, PMID: 38277314

[9] Liu Z Zhang X Yu B Wang J Lu X. Effectiveness on level of consciousness of non-invasive neuromodulation therapy in patients with disorders of consciousness: a systematic review and meta-analysis. Front Hum Neurosci. (2023) 17:1129254. doi: 10.3389/fnhum.2023.1129254, PMID: 37292582 PMC10246452

[10] Jung IH Chang KW Park SH Chang WS Jung HH Chang JW. Complications after deep brain stimulation: a 21-year experience in 426 patients. Front Aging Neurosci. (2022) 14:819730. doi: 10.3389/fnagi.2022.819730, PMID: 35462695 PMC9022472

[11] Dong L Li H Dang H Zhang X Yue S Zhang H. Efficacy of non-invasive brain stimulation for disorders of consciousness: a systematic review and meta-analysis. Front Neurosci. (2023) 17:1219043. doi: 10.3389/fnins.2023.1219043, PMID: 37496734 PMC10366382

[12] Martens G Lejeune N O’Brien AT Fregni F Martial C Wannez S . Randomized controlled trial of home-based 4-week tDCS in chronic minimally conscious state. Brain Stimul. (2018) 11:982–90. doi: 10.1016/j.brs.2018.04.021, PMID: 29759943

[13] Jia J. Exploration on neurobiological mechanisms of the central-peripheral-central closed-loop rehabilitation. Front Cell Neurosci. (2022) 16:982881. doi: 10.3389/fncel.2022.982881, PMID: 36119128 PMC9479450

[14] Xiong Q Le K Tang Y Ye W Wang Y Zhong Y . Effect of single and combined median nerve stimulation and repetitive transcranial magnetic stimulation in patients with prolonged disorders of consciousness: a prospective, randomized, single-blinded, controlled trial. Front Aging Neurosci. (2023) 15:1112768. doi: 10.3389/fnagi.2023.1112768, PMID: 37168716 PMC10164991

[15] Almalty AR Hamed SH Jebril MY Abdelnour HM. The effect of electrical stimulation on skin vulnerability to irritants. Skin Res Technol. (2024) 30:e13591. doi: 10.1111/srt.13591, PMID: 38279544 PMC10818122

[16] Reddy J Singhal R Gaikwad AP Patel D Patel P Gandhi SK. Unraveling the potential of electroanalgesia: a literature review of current therapeutics. Cureus. (2024) 16:e61122. doi: 10.7759/cureus.61122, PMID: 38919207 PMC11198869

[17] Koch G Esposito R Motta C Casula EP Di Lorenzo F Bonnì S . Improving visuo-motor learning with cerebellar theta burst stimulation: behavioral and neurophysiological evidence. NeuroImage. (2020) 208:116424. doi: 10.1016/j.neuroimage.2019.116424, PMID: 31794855

[18] Jia Y Liu X Wei J Li D Wang C Wang X . Modulation of the corticomotor excitability by repetitive peripheral magnetic stimulation on the median nerve in healthy subjects. Front Neural Circuits. (2021) 15:616084. doi: 10.3389/fncir.2021.616084, PMID: 33815069 PMC8012681

[19] Park S Park R Westwood D Moayedi M Khan JS. Effect of peripheral magnetic stimulation on acute and chronic pain after surgery: a systematic review and meta-analysis. J Pain. (2023) 24:1151–62. doi: 10.1016/j.jpain.2023.02.031, PMID: 36878385

[20] Giacino JT Kalmar K Whyte J. The JFK coma recovery scale-revised: measurement characteristics and diagnostic utility. Arch Phys Med Rehabil. (2004) 85:2020–9. doi: 10.1016/j.apmr.2004.02.033, PMID: 15605342

[21] Seel RT Sherer M Whyte J Katz DI Giacino JT Rosenbaum AM . Assessment scales for disorders of consciousness: evidence-based recommendations for clinical practice and research. Arch Phys Med Rehabil. (2010) 91:1795–813. doi: 10.1016/j.apmr.2010.07.218, PMID: 21112421

[22] Zorumski CF Mennerick S Izumi Y. Acute and chronic effects of ethanol on learning-related synaptic plasticity. Alcohol. (2014) 48:1–17. doi: 10.1016/j.alcohol.2013.09.045, PMID: 24447472 PMC3923188

[23] Rogawski MA. Update on the neurobiology of alcohol withdrawal seizures. Epilepsy Curr. (2005) 5:225–30. doi: 10.1111/j.1535-7511.2005.00071.x, PMID: 16372057 PMC1312739

[24] Sullivan EV Pfefferbaum A. Neurocircuitry in alcoholism: a substrate of disruption and repair. Psychopharmacology. (2005) 180:583–94. doi: 10.1007/s00213-005-2267-6, PMID: 15834536

[25] Yang Z Yue T Zschorlich VR Li D Wang D Qi F. Behavioral effects of repetitive transcranial magnetic stimulation in disorders of consciousness: a systematic review and Meta-analysis. Brain Sci. (2023) 13:1362. doi: 10.3390/brainsci13101362, PMID: 37891731 PMC10605911

[26] Monti MM Spivak NM Edlow BL Bodien YG. What is a minimal clinically important difference for clinical trials in patients with disorders of consciousness? A novel probabilistic approach. PLoS One. (2023) 18:e0290290. doi: 10.1371/journal.pone 37616196 PMC10449161

[27] Huang W Chen Q Liu J Liu L Tang J Zou M . Transcranial magnetic stimulation in disorders of consciousness: an update and perspectives. Aging Dis. (2023) 14:01–1183. doi: 10.14336/AD.2022.1114, PMID: 37163434 PMC10389824

[28] Thibaut A Bruno MA Ledoux D Demertzi A Laureys S. Tdcs in patients with disorders of consciousness: sham-controlled randomized double-blind study. Neurology. (2014) 82:1112–8. doi: 10.1212/WNL.0000000000000260, PMID: 24574549

[29] Bai Y Xia X Li X Wang Y Yang Y Liu Y . Spinal cord stimulation modulates frontal delta and gamma in patients of minimally consciousness state. Neuroscience. (2017) 346:247–54. doi: 10.1016/j.neuroscience.2017.01.036, PMID: 28147246

[30] He F Wu M Meng F Hu Y Gao J Chen Z . Effects of 20 Hz repetitive transcranial magnetic stimulation on disorders of consciousness: a resting-state electroencephalography study. Neural Plast. (2018) 2018:5036184. doi: 10.1155/2018/5036184, PMID: 29770146 PMC5889874

[31] Rohaut B Calligaris C Hermann B Perez P Faugeras F Raimondo F . Multimodal assessment improves neuroprognosis performance in clinically unresponsive critical-care patients with brain injury. Nat Med. (2024) 30:2349–55. doi: 10.1038/s41591-024-03019-1, PMID: 38816609 PMC11333287

[32] Rossi S Hallett M Rossini PM Pascual-Leone A Safety of TMS Consensus Group. Safety, ethical considerations, and application guidelines for the use of transcranial magnetic stimulation in clinical practice and research. Clin Neurophysiol. (2009) 120:2008–39. doi: 10.1016/j.clinph.2009.08.016, PMID: 19833552 PMC3260536

[33] Rossi S Antal A Bestmann S Bikson M Brewer C Brockmöller J . Safety and recommendations for TMS use in healthy subjects and patient populations, with updates on training, ethical and regulatory issues: expert guidelines. Clin Neurophysiol. (2021) 132:269–306. doi: 10.1016/j.clinph.2020.10.003, PMID: 33243615 PMC9094636

[34] Edlow BL Claassen J Schiff ND Greer DM. Recovery from disorders of consciousness: mechanisms, prognosis and emerging therapies. Nat Rev Neurol. (2021) 17:135–56. doi: 10.1038/s41582-020-00428-x, PMID: 33318675 PMC7734616

[35] van der Jagt M Robba C Skrifvars MB. Unlocking consciousness through right median nerve stimulation. Has a potential cure arrived at our doorstep? Intensive Care Med. (2023) 49:659–61. doi: 10.1007/s00134-023-07097-6, PMID: 37210686 PMC10199817

[36] Badcock PB Friston KJ Ramstead MJD. The hierarchically mechanistic mind: a free-energy formulation of the human psyche. Phys Life Rev. (2019) 31:104–21. doi: 10.1016/j.plrev.2018.10.002, PMID: 30704846 PMC6941235

[37] Fischer DB Boes AD Demertzi A Evrard HC Laureys S Edlow BL . A human brain network derived from coma-causing brainstem lesions. Neurology. (2016) 87:2427–34. doi: 10.1212/WNL.0000000000003404, PMID: 27815400 PMC5177681

[38] Qi F Nitsche MA Ren X Wang D Wang L. Top-down and bottom-up stimulation techniques combined with action observation treatment in stroke rehabilitation: a perspective. Front Neurol. (2023) 14:1156987. doi: 10.3389/fneur.2023.1156987, PMID: 37497013 PMC10367110

[39] Nardone R Höller Y Langthaler PB Lochner P Golaszewski S Schwenker K . rTMS of the prefrontal cortex has analgesic effects on neuropathic pain in subjects with spinal cord injury. Spinal Cord. (2017) 55:20–5. doi: 10.1038/sc.2016.87, PMID: 27241450

[40] Moretti J Terstege DJ Poh EZ Epp JR Rodger J. Low intensity repetitive transcranial magnetic stimulation modulates brain-wide functional connectivity to promote anti-correlated c-Fos expression. Sci Rep. (2022) 12:20571–82. doi: 10.1038/s41598-022-24934-8, PMID: 36446821 PMC9708643

[41] Hansen JY Cauzzo S Singh K García-Gomar MG Shine JM Bianciardi M . Integrating brainstem and cortical functional architectures. Nat Neurosci. (2024) 27:2500–11. doi: 10.1038/s41593-024-01787-0, PMID: 39414973 PMC11614745

[42] Fitzsimmons SMDD Oostra E Postma TS van der Werf YD van den Heuvel OA. Repetitive transcranial magnetic stimulation-induced neuroplasticity and the treatment of psychiatric disorders: state of the evidence and future opportunities. Biol Psychiatry. (2024) 95:592–600. doi: 10.1016/j.biopsych.2023.11.016, PMID: 38040046

[43] Maegaki Y Najm I Terada K Morris HH Bingaman WE Kohaya N . Somatosensory evoked high-frequency oscillations recorded directly from the human cerebral cortex. Clin Neurophysiol. (2000) 111:1916–26. doi: 10.1016/s1388-2457(00)00449-1, PMID: 11068223

[44] Keep RF Hua Y Xi G. Intracerebral haemorrhage: mechanisms of injury and therapeutic targets. Lancet Neurol. (2012) 11:720–31. doi: 10.1016/S1474-4422(12)70104-7, PMID: 22698888 PMC3884550

[45] Geocadin RG Wijdicks E Armstrong MJ Damian M Mayer SA Ornato JP . Practice guideline summary: reducing brain injury following cardiopulmonary resuscitation: report of the guideline development, dissemination, and implementation Subcommittee of the American Academy of neurology. Neurology. (2017) 88:2141–9. doi: 10.1212/WNL.0000000000003966, PMID: 28490655 PMC5447399

[46] Fecteau S Agosta S Hone-Blanchet A Fregni F Boggio P Ciraulo D . Modulation of smoking and decision-making behaviors with transcranial direct current stimulation in tobacco smokers: a preliminary study. Drug Alcohol Depend. (2014) 140:78–84. doi: 10.1016/j.drugalcdep.2014.03.036, PMID: 24814566 PMC4242508

[47] Naro A Bramanti P Leo A Russo M Calabrò RS. Transcranial alternating current stimulation in patients with chronic disorder of consciousness: a possible way to cut the diagnostic Gordian knot? Brain Topogr. (2016) 29:623–44. doi: 10.1007/s10548-016-0489-z, PMID: 27062669

[48] Legostaeva L Poydasheva A Iazeva E Sinitsyn D Sergeev D Bakulin I . Stimulation of the angular gyrus improves the level of consciousness. Brain Sci. (2019) 9:103–15. doi: 10.3390/brainsci9050103, PMID: 31064138 PMC6562708

[49] Fuller PM Sherman D Pedersen NP Saper CB Lu J. Reassessment of the structural basis of the ascending arousal system. J Comp Neurol. (2011) 519:933–56. doi: 10.1002/cne.22559, PMID: 21280045 PMC3119596

